# Connective Tissue Disease-Associated Pulmonary Arterial Hypertension: Current Therapeutic Strategies and Future Prospects

**DOI:** 10.3390/biom16010140

**Published:** 2026-01-13

**Authors:** Yukina Mizuno Yokoyama, Ryu Watanabe, Tomohiro Yamaguchi, Ryuhei Ishihara, Mayu Shiomi, Yuya Fujita, Masao Katsushima, Kazuo Fukumoto, Yoichiro Haji, Shinsuke Yamada, Motomu Hashimoto

**Affiliations:** 1 Department of Clinical Immunology, Osaka Metropolitan University Graduate School of Medicine, 1-4-3, Asahi-machi, Abeno-Ku, Osaka 545-8585, Japan; 2 Department of Rheumatology, Daido Hospital, Nagoya 457-8511, Japan; 3 Department of Cardiovascular Medicine, Osaka Metropolitan University Graduate School of Medicine, Osaka 545-8585, Japan

**Keywords:** animal model, biomarker, connective tissue disease, pulmonary arterial hypertension, systemic sclerosis

## Abstract

Connective tissue disease-associated pulmonary arterial hypertension (CTD-PAH) is a severe form of pulmonary hypertension with poor prognosis. It most commonly arises in systemic sclerosis (SSc), followed by systemic lupus erythematosus (SLE) and mixed connective tissue disease (MCTD). Its pathogenesis involves a complex interplay of immune dysregulation, chronic inflammation, endothelial injury, vascular remodeling, and fibrosis. Although vasodilators targeting the endothelin, nitric oxide, and prostacyclin pathways remain the therapeutic backbone, newer agents—including the activin signal inhibitor sotatercept and inhaled treprostinil—have expanded treatment options. Immune-targeted therapies such as glucocorticoids, cyclophosphamide, mycophenolate mofetil, rituximab, and IL-6 receptor inhibitors may benefit inflammation-dominant PAH phenotypes, while fibrotic phenotypes continue to demonstrate limited responsiveness. In addition to brain natriuretic peptide (BNP), N-terminal (NT)-proBNP and disease-specific autoantibodies, emerging biomarkers show promise for early detection, risk stratification, and personalized treatment, though validation in CTD-PAH is lacking. Advances in animal models replicating immune-mediated vascular injury and fibrosis have further improved mechanistic understanding. Despite these developments, substantial unmet needs remain, including the absence of disease-specific therapeutic strategies, limited biomarker integration into clinical practice, and a scarcity of large, well-designed trials targeting individual CTD subtypes. Addressing these gaps will be essential for improving prognosis in patients with CTD-PAH.

## 1. Introduction

Pulmonary hypertension is a life-threatening condition characterized by elevated pulmonary arterial pressure and increased pulmonary vascular resistance due to loss of the pulmonary vascular bed and obstructive vascular remodeling, ultimately progressing to right ventricular failure [1]. It is currently estimated to affect approximately 1% of the global population, with prevalence rising further in the presence of comorbid cardiopulmonary diseases, particularly among older adults [2].

Clinically, pulmonary hypertension is classified into five groups (groups I–V). Worldwide, left heart disease (group II) represents the most common etiology, followed by pulmonary disorders such as chronic obstructive pulmonary disease (group III). Pulmonary arterial hypertension (PAH, group I) is relatively rare, with an incidence of 48 to 55 cases per million individuals [2], and within this group, idiopathic PAH is the most prevalent subtype followed by connective tissue disease-associated PAH (CTD-PAH) [2]. Group IV includes chronic thromboembolic pulmonary hypertension (CTEPH) and other pulmonary artery obstructive disorders, whereas Group V encompasses pulmonary hypertension associated with hematologic diseases and other multifactorial or unclear mechanisms; both groups are considered rare.

For an accurate etiological diagnosis of pulmonary hypertension, a comprehensive diagnostic approach is required, integrating transthoracic echocardiography, pulmonary function testing, high-resolution computed tomography of the chest, ventilation–perfusion scintigraphy, and right heart catheterization. In clinical practice, however, distinguishing PAH (Group I) from pulmonary hypertension due to left heart disease (Group II) can be challenging, particularly in patients with borderline hemodynamic findings, in whom fluid challenge with isotonic saline during right heart catheterization may be useful [3]. Likewise, differentiation between pulmonary hypertension associated with lung diseases (Group III) and PAH with coexisting mild pulmonary disease (Group I) is frequently encountered in daily practice [4]. In addition, several rare conditions, including pulmonary artery sarcoma, fibrosing mediastinitis, and pulmonary sarcoidosis, have been reported to mimic CTEPH, further complicating the diagnostic process [5].

In recent years, the hemodynamic definition of pulmonary hypertension has been revised, lowering the diagnostic threshold for mean pulmonary arterial pressure (mPAP) from ≥25 mmHg to >20 mmHg and introducing pulmonary vascular resistance (PVR) >2 Wood units as an additional criterion [2,6]. Following this redefinition, cases with mildly elevated mPAP (21–24 mmHg), previously considered within a borderline range, have gained clinical attention, with increasing interest in the potential benefits of early therapeutic intervention.

CTD-PAH is increasingly recognized as a heterogeneous disease entity encompassing diverse underlying mechanisms and clinical phenotypes. This heterogeneity poses significant challenges for diagnosis, risk stratification, and therapeutic decision-making, underscoring the need for an integrated, mechanism-based framework to contextualize current evidence and guide clinical management.

In the following sections, CTD-PAH is discussed in a disease-specific manner, with attention to the distinct epidemiological and pathological characteristics of each disease (Table 1).

## 2. Epidemiology and Pathology of CTD-PAH

### 2.1. Pathogenic Mechanisms Underlying CTD-PAH

Recent advances have reshaped the understanding of CTD-PAH as a heterogeneous disease spectrum rather than a single pathological entity. Accumulating evidence supports a conceptual continuum ranging from inflammation-dominant, immune-mediated phenotypes to fibrosis-dominant phenotypes characterized by irreversible vascular remodeling. In the early or immune-dominant stage—exemplified by systemic lupus erythematosus-associated PAH (SLE-PAH) and a subset of mixed connective tissue disease-associated PAH (MCTD-PAH)—dysregulated innate and adaptive immune responses play a central role. Activated T cells, B cells, and macrophages infiltrate the pulmonary vasculature and produce pro-inflammatory cytokines, including IL-6, IL-17, TNF-α, and type I interferons. These mediators induce endothelial dysfunction, promote apoptosis-resistant endothelial phenotypes, and disrupt vasodilatory signaling, thereby initiating pulmonary vascular injury.

With persistent immune activation, chronic inflammation transitions toward a fibrosis-dominant stage, which is most prominently observed in systemic sclerosis-associated PAH (SSc-PAH). Profibrotic signaling pathways, particularly those involving transforming growth factor-β (TGF-β), activins, and SMAD2/3, become dominant, leading to smooth muscle cell proliferation, extracellular matrix deposition, and progressive luminal narrowing. Concurrently, disruption of bone morphogenetic protein (BMP) signaling—often associated with reduced BMPR2 activity—further accelerates maladaptive vascular remodeling.

Importantly, the relative contribution of immune-mediated inflammation and fibrotic remodeling varies across CTD subtypes and disease stages, accounting for the marked heterogeneity in treatment responsiveness. This evolving mechanistic framework provides a biological rationale for phenotype-oriented therapeutic strategies, including the preferential use of immunosuppressive therapy in inflammation-dominant CTD-PAH and anti-remodeling approaches in fibrosis-dominant disease.

**Figure 1:**
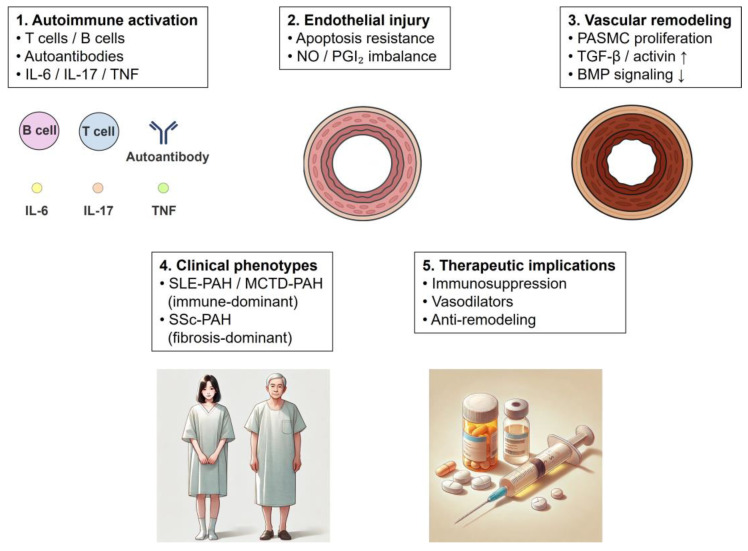
Schematic overview of the pathogenic continuum in connective tissue disease-associated pulmonary arterial hypertension (CTD-PAH). Autoimmune activation induces immune-mediated endothelial injury through inflammatory cytokines and autoantibodies, leading to dysregulated vasodilatory signaling. Persistent inflammation promotes vascular remodeling characterized by pulmonary arterial smooth muscle cell (PASMC) proliferation and extracellular matrix deposition via profibrotic pathways. The relative contribution of immune-driven inflammation and fibrotic remodeling varies across CTD subtypes, resulting in immune-dominant phenotypes such as systemic lupus erythematosus-associated PAH (SLE-PAH) and a subset of mixed connective tissue disease-associated PAH (MCTD-PAH), and fibrosis-dominant phenotypes such as systemic sclerosis-associated PAH (SSc-PAH). These mechanistic differences provide a biological rationale for phenotype-oriented therapeutic strategies.

These pathogenic processes and their interrelationships are schematically summarized in Figure 1.

### 2.2. Systemic Sclerosis (SSc-PAH)

Systemic sclerosis (SSc) is the most common underlying condition among CTD-PAH, accounting for approximately 74% of all CTD-PAH cases in European and North American registries [7]. The prevalence of PAH among patients with SSc is reported to be 7–19% overall [7,8], including 7.7% in limited cutaneous SSc (lcSSc) and 6.3% in diffuse cutaneous SSc (dcSSc) [9]. Although the frequency tends to be slightly higher in lcSSc, the difference from dcSSc is relatively small [10]. Recent revisions to the hemodynamic definition have led to the identification of additional mild cases, including those with mPAP 21–24 mmHg; however, the increase has been modest (approximately 1–2%), and only a subset of previously latent early-stage cases has become clinically apparent [11].

SSc-associated PAH (SSc-PAH) exhibits the poorest treatment response among CTD-PAH subtypes, with outcomes characterized by a 5-year survival rate of 42–61.9% and a 10-year survival rate of approximately 26.8% [12,13]. This unfavorable prognosis is attributable to an initial pathological process driven by autoimmune-mediated chronic inflammation, resulting in endothelial injury that progresses to irreversible vascular remodeling and fibrosis. Inflammatory cytokines produced by activated T cells and B cells promote intimal proliferation, medial thickening, vascular luminal narrowing, and microthrombus formation, ultimately leading to right ventricular failure. Given that the relative contribution of these pathogenic mechanisms varies among individual patients—and that pulmonary hypertension secondary to left heart disease (Group II) or interstitial lung disease (ILD) (Group III) may also coexist—the heterogeneity in underlying physiology results in widely differing treatment responsiveness, further contributing to the poor overall prognosis observed in SSc-PAH [14,15]. In addition, recent evidence indicates that genetic and molecular factors—including HLA polymorphisms and cytokine-related gene variants—also contribute to disease susceptibility and progression [16].

Well-established predictors of poor prognosis include male sex, advanced age, concomitant ILD, and impaired right ventricular function [7]. Analyses from the EUSTAR cohort have further demonstrated that reduced diffusing capacity for carbon monoxide (DLCO) serves as an important independent prognostic indicator [17]. Recent studies have shown that patients whose PAH is detected through proactive screening have significantly better prognoses than those diagnosed after the onset of symptoms [18]. Accordingly, annual screening with transthoracic echocardiography and measurement of BNP or NT-proBNP is recommended.

### 2.3. Systemic Lupus Erythematosus (SLE-PAH)

Systemic lupus erythematosus-associated pulmonary arterial hypertension (SLE-PAH) accounts for approximately 8% of CTD-PAH cases in Western registries [7], making it the second most frequent subtype after SSc-PAH. In contrast, reports from Asian countries indicate that SLE-PAH constitutes more than half (57%) of CTD-PAH cases, highlighting substantial geographical variability [19]. The reported prevalence of PAH among patients with SLE ranges widely, from 0.5% to 17.5% [20,21].

The prognosis of SLE-PAH is comparatively favorable among CTD-PAH subtypes, with 1-, 3-, and 5-year survival rates estimated at approximately 88%, 81%, and 68%, respectively [22]. This relatively good prognosis is thought to reflect an underlying pathophysiology driven mainly by reversible immune dysregulation and inflammation—such as immune complex deposition, complement activation, and vasculitis—rendering early immunosuppressive therapy more likely to yield benefit [23,24]. Moreover, recent studies have identified gene expression signatures shared between SLE and PAH, implicating hyperactivation of type I interferon (IFN) signaling as a common pathogenic pathway [25]. These findings further support the rationale for immune-targeted therapeutic approaches in SLE-PAH.

### 2.4. Mixed Connective Tissue Disease (MCTD-PAH)

Mixed connective tissue disease (MCTD) is a relatively uncommon condition, accounting for approximately 8% of all CTD-PAH cases [7], making it the third most frequent subtype following SSc and SLE. In the 1980s, the prevalence of PAH among patients with MCTD was estimated to be as high as 29%; however, more recent studies report a substantially lower prevalence of approximately 12.5% [26], suggesting that earlier diagnosis and the introduction of immunosuppressive therapy may have contributed to this decline.

Despite its relative rarity, PAH in MCTD can lead to severe clinical consequences. Some studies have documented, in patients with MCTD, pulmonary hypertension has been reported to contribute to up to 81.8% of all deaths [27], indicating that the timing of immunosuppressive treatment plays a critical role in determining clinical outcomes. The 5-year survival rate is reported to be approximately 59% [13]. While patients with predominantly inflammatory pathology may respond favorably to therapy, treatment resistance often emerges once irreversible fibrotic vascular remodeling has developed. Importantly, MCTD encompasses a spectrum of phenotypes, and SSc-like PAH—characterized by prominent fibrosis—tends to exhibit poor treatment responsiveness, whereas SLE-like PAH generally shows a more favorable response to immunosuppressive therapy; thus, distinguishing between these phenotypic patterns is crucial for appropriate therapeutic decision-making.

### 2.5. Rheumatoid Arthritis (RA-PAH)

PAH associated with rheumatoid arthritis (RA) is extremely rare, with less than 1% of cases confirmed by right heart catheterization [28]. In contrast, echocardiography-based studies have reported incidences exceeding 10% [28]; however, these findings are thought to predominantly reflect pulmonary hypertension due to comorbid conditions—most commonly left-sided heart failure (group II) and interstitial lung disease (group III) [29].

With regard to treatment, there is limited evidence to suggest that immunosuppressive therapy directly ameliorates pulmonary vascular remodeling in RA-associated PAH. Therefore, comprehensive assessment and management of accompanying cardiopulmonary comorbidities are clinically essential in the care of these patients.

### 2.6. Sjögren Disease (SjD-PAH)

Sjögren disease-associated PAH (SjD-PAH), formerly referred to as Sjögren syndrome [30], is relatively rare, with a reported prevalence of approximately 1.6% based on right heart catheterization data [31]. The condition is reported to occur more frequently in younger women and in patients with a relatively short disease duration [32].

Patients who are positive for anti-U1RNP antibodies have a markedly increased risk of developing PAH (odds ratio 29.5), suggesting a pathophysiology that may resemble that of MCTD [33]. The 5-year survival rate is estimated to be approximately 79.0%, which is comparatively favorable [33].

### 2.7. Idiopathic Inflammatory Myopathy (IIM-PAH)

Idiopathic inflammatory myopathy-associated PAH (IIM-PAH) is exceedingly rare. In the French PH registry, PAH was confirmed in only 3 of 5223 patients with IIM, all of whom had dermatomyositis [34]. Other studies similarly indicate that most cases of pulmonary hypertension in IIM belong to group 3 and are attributable to interstitial lung disease, whereas Group I PAH accounts for only approximately 30% of cases [35].

The underlying pathophysiology is thought to involve subacute or chronic pulmonary microangiopathy resulting from pulmonary vascular endothelial injury and autoimmune dysregulation [34]. Risk factors associated with disease development include anti–SS-A antibody positivity, high inflammatory disease activity, Raynaud’s phenomenon, and nailfold capillary changes (NFCC) [34]. Notably, the dermatomyositis phenotype appears to confer a particularly elevated risk. The prognosis is relatively favorable, with 5-year survival rates reported to range from 91.7% to 94.4% [36].

### 2.8. Takayasu Arteritis (TAK-PH)

Takayasu’s arteritis (TAK) is a form of large-vessel vasculitis, and pulmonary artery involvement can occur in up to half of affected patients. However, only 5–15% of all TAK cases progress to PH as defined hemodynamically, and most such cases arise from stenosis or occlusion of the pulmonary artery rather than from intrinsic pulmonary vascular remodeling. Consequently, the pathophysiology of TAK-associated PH is considered distinct from that of typical Group 1 PAH [37]. Therefore, TAK-PAH needs to be understood as a separate pathological condition of outflow tract stenosis associated with macrovascular lesions, rather than pulmonary vascular remodeling.

The main treatment is based on immunosuppressive therapy for TAK, but in cases with pulmonary artery stenosis, endovascular treatments such as percutaneous pulmonary angioplasty are used in combination [38].

**Table 1:** Summary of representative cohort studies reporting the prevalence, prognosis, and risk factors of PAH in each underlying connective tissue disease.

	Proportion Among CTD-PAH	Prevalence of PH	Overall Survival	Risk Factors for Pulmonary Hypertension	Region/Country	References
Systemic sclerosis (SSc-PAH)	74%	8~12%	1–2 years 35–45% (untreated)	Older age at onset, Longer disease duration, limited type, low DLCO, positive anticentromere antibodies, telangiectasia, male	Europe (Italy)	[4]
	64%	7.5–12%	1 year 85% 3 year 59% 5 year 42%	N/A	Europe: COMPERA registry	[9]
	7.9%	N/A	1 year 85.0% 3 year 65.0% 5 year 53.6% 10 year 26.8%	N/A	Asia (China)	[8]
Systemic lupus erythematosus (SLE-PAH)	8%	1–5%	N/A	Anticardiolipin antibodies, lupus anticoagulant, anti-U1RNP antibodies	Europe (Italy)	[4]
	5.6%	N/A	1 year 97% 3 year 77% 5 year 61%	N/A	Europe: COMPERA registry	[9]
	N/A	2.13%	1 year 87.7% 3 year 76.8% 5 year 70.1%	Hypertension	Asia (Taiwan)	[16]
	N/A	N/A	1 year 90.0% 3 year 80.3% 5 year 73.8% 10 year 64.3%	N/A	Asia (China)	[8]
	N/A	1.5%	N/A	Low DLCO Elevated NT-proBNP	Europe (Spain)	[17]
Mixed Connective Tissue Disease (MCTD-PAH)	8%	3–4%	N/A	SSc-like symptom (e.g., skin sclerosis)	Europe (Italy)	[4]
	4.1%	N/A	1 year 97% 3 year 70% 5 year 59%	N/A	Europe: COMPERA registry	[9]
	N/A	N/A	1 year 92.9% 3 year 89.3% 5 year 81.5%	N/A	Asia (China)	[8]
Rheumatoid arthritis (RA-PAH)	N/A	Very rare	N/A	N/A	Europe (Italy)	[4]
	1.6%	N/A	1 year 100% 3 year 88.9% 5 year 44.4%	N/A	Europe: COMPERA registry	[9]
	N/A	N/A	1 year 100% 3 year 100% 5 year 72.2%	N/A	Asia (China)	[8]
Sjögren disease (SjD-PAH)	N/A	Very rare	N/A	N/A	Europe (Italy)	[4]
	0.8%	N/A	N/A	N/A	Europe: COMPERA registry	[9]
	N/A	Very rare	1 year 94.0% 3 year 88.8% 5 year 79.0% 10 year 67.2%	Anti-SSB antibody, Anti-U1RNP antibody, Early age at onset, Corneal damage		[29]
Idiopathic inflammatory myopathy (IIM-PAH)	N/A	Rare	N/A	Dermatomyositis type Peripheral vascular disease	Europe (Italy)	[4]
	N/A	N/A	N/A	Anti-SS-A antibody, High disease activity, Raynaud phenomenon, NFCC	Europe (France)	[30]
	N/A	N/A	5 year 91.7~94.4%	N/A	Africa (Oman)	[32]
Takayasu’s arteritis (TAK-PAH)	N/A	8.5% (PH)	N/A	Pulmonary artery involvement	Asia (China)	[33]

CTD, connective tissue disease; DLCO, diffusing capacity for carbon monoxide; N/A, not available; NFCC, nailfold capillary changes; PAH, pulmonary arterial hypertension; PH, pulmonary hypertension.

## 3. Risk Stratification and Treatment Goals in CTD-PAH

Risk stratification has become a central component of PAH management, providing prognostic information and guiding therapeutic decision-making at baseline and during follow-up. Current international guidelines recommend a multidimensional risk assessment approach incorporating World Health Organization functional class (WHO-FC), 6 min walk distance (6 MWD), and circulating natriuretic peptides, including BNP and NT-proBNP, with the overarching therapeutic objective of achieving and sustaining a low-risk status [39,40].

However, the applicability of existing risk stratification models to CTD-PAH, particularly SSc-PAH, remains challenging. In these patients, exercise capacity and biomarkers may be substantially influenced by coexisting pulmonary disease, as well as extrapulmonary manifestations such as musculoskeletal involvement, left heart dysfunction, or renal impairment, potentially leading to misclassification of disease severity.

Recent studies have emphasized the importance of incorporating markers of right ventricular function and pulmonary vascular remodeling—including echocardiographic parameters, cardiac magnetic resonance imaging, and invasive hemodynamics—to refine risk assessment, particularly in patients categorized as intermediate risk. Serial risk assessment during follow-up appears to provide greater prognostic value than baseline evaluation alone and may better reflect treatment response [39,41].

Importantly, in CTD-PAH, risk stratification should not be interpreted in isolation but rather in the context of underlying disease phenotype. Patients with inflammation-dominant disease may exhibit reversible hemodynamic abnormalities and greater responsiveness to immunosuppressive therapy, whereas those with fibrosis-dominant vascular remodeling often demonstrate limited reversibility despite aggressive vasodilator treatment. Integrating clinical risk scores with phenotypic markers of immune activation and fibrotic burden may therefore provide a more clinically meaningful framework for therapeutic decision-making.

## 4. Current Therapies and Emerging Therapeutic Agents in PAH

### 4.1. Vasodilators Targeting Three Major Pathways

Current standard therapy for PAH is based on vasodilators that target three major signaling pathways: the endothelin (ET) pathway, the nitric oxide (NO) pathway, and the prostacyclin (PGI2) pathway [42].

#### 4.1.1. Endothelin Pathway

**Figure 2:**
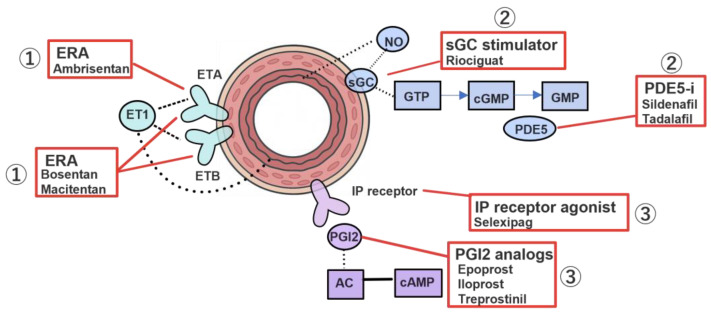
Vasodilators targeting three major pathways. In pulmonary arterial hypertension, an imbalance between vasoconstriction and vasodilation arises from the excessive expression of the vasoconstrictor endothelin-1 (ET-1) and the diminished expression of the vasodilatory enzymes nitric oxide (NO) synthase and prostacyclin (PGI_2_) synthase. ① Endothelin receptor antagonists (ERAs) inhibit ET-1–mediated vasoconstrictive signaling. Ambrisentan selectively blocks ETA receptors, whereas bosentan and macitentan antagonize both ETA and ETB receptors. ② Agents targeting the NO pathway include phosphodiesterase type 5 (PDE5) inhibitors (sildenafil, tadalafil), which prevent the degradation of cyclic guanosine monophosphate (cGMP), and the soluble guanylate cyclase (sGC) stimulator riociguat, which enhances cGMP synthesis. ③ Prostacyclin-pathway agents, such as PGI_2_ analogs (epoprostenol, iloprost, treprostinil) and the IP receptor agonist selexipag, promote vasodilation.

Endothelin-1 (ET-1) is a potent vasoconstrictor and vascular smooth muscle cell mitogen that exerts its effects through the ETA and ETB receptors (Figure 2). Therapeutic agents targeting this pathway include selective ETA receptor antagonists as well as dual ETA/ETB receptor antagonists, both of which contribute to the treatment of PAH by inhibiting vasoconstriction and vascular smooth muscle proliferation [42].

In the SERAPHIN trial, macitentan reduced the risk of death or clinical worsening by 45% (hazard ratio [HR] 0.55, *p* < 0.001) [43]. In Japan, macitentan has been the most widely used endothelin receptor antagonist among patients with CTD-PAH, according to the JAPHR registry [44]. Macitentan often plays a central role in triple combination therapy targeting three major pathways. Additionally, ambrisentan was shown to improve 6 MWD and decrease the risk of clinical deterioration in the ARIES trials [45]. More recently, the 2024 EDITA trial—which enrolled patients with mean pulmonary artery pressure (mPAP) of 21–24 mmHg—demonstrated sustained therapeutic benefit with long-term administration (mean 2.6 years) [46].

Endothelin receptor antagonists are generally well tolerated; however, clinically relevant adverse effects include hepatotoxicity, fluid retention, peripheral edema, anemia, and teratogenicity. Therefore, regular monitoring of liver function tests and hemoglobin levels is required, and their use is contraindicated during pregnancy.

#### 4.1.2. Nitric Oxide (NO) Pathway

Nitric oxide (NO) activates soluble guanylyl cyclase (sGC), leading to vasodilation through increased cyclic guanosine monophosphate (cGMP) production (Figure 2). Phosphodiesterase type 5 (PDE5) inhibitors prevent the degradation of cGMP, whereas sGC stimulators enhance this pathway by promoting cGMP synthesis [42].

In the AMBITION trial, initial combination therapy with ambrisentan and tadalafil (a PDE5 inhibitor) in treatment-naïve patients with PAH reduced the risk of clinical worsening by approximately 50% [6]. Based on these findings, the ESC/ERS guidelines now recommend upfront combination therapy [2]. Furthermore, in 2024, the efficacy and safety of this combination regimen were reported regardless of the presence or absence of cardiovascular comorbidities [47]. Notably, the AMBITION trial included 187 patients with CTD-PAH, of whom 118 had SSc-PAH. In these subgroups, initial combination therapy with ambrisentan and tadalafil significantly reduced the risk of clinical failure compared with monotherapy, with hazard ratios of 0.43 (95% CI 0.24–0.77) for CTD-PAH and 0.44 (95% CI 0.22–0.89) for SSc-PAH [48].

In addition, the PATENT-1 and PATENT-2 trials demonstrated that the sGC stimulator riociguat improved mean 6 MWD, WHO functional class, pulmonary vascular resistance and cardiac index in patients with CTD-PAH [49]. Moreover, switching from PDE5i to riociguat resulted in clinically meaningful improvement in patients who failed to achieve treatment targets with PDE5 inhibitors alone or in combination with endothelin receptor antagonists [50]. However, in idiopathic interstitial pneumonia-associated pulmonary hypertension, riociguat was associated with increased all-cause mortality and a higher incidence of serious respiratory adverse events, leading to an unfavorable risk–benefit balance [51]. Therefore, the use of riociguat in CTD-PAH patients with concomitant ILD requires particular caution.

Agents targeting the NO pathway, including PDE5i and sGC stimulators, are generally well tolerated. Common adverse effects include headache, flushing, nasal congestion, dyspepsia, hypotension, and dizziness. Concomitant use with nitrates is contraindicated because of the risk of severe hypotension.

Beyond their pharmacological modulation by PDE5i and sGC stimulators, nitric oxide (NO) and carbon monoxide (CO) constitute a broader guanylate-centered signaling axis that plays a fundamental role in pulmonary vascular homeostasis [52]. Binding of CO to sGC, analogous to that of NO, results in enzyme activation, subsequent cGMP production, and inhibition of vascular smooth muscle cell proliferation [53,54]. Although CO exerts systemic and cellular toxicity at high concentrations, it has been shown to confer cytoprotective and tissue-protective effects at low concentrations in experimental models of organ injury and disease. Dysregulation of CO–cGMP signaling may therefore represent an important pathogenic mechanism in CTD-PAH.

#### 4.1.3. Prostacyclin (PGI_2_) Pathway

Prostacyclin (PGI_2_) exerts potent vasodilatory and antiproliferative effects via activation of the prostacyclin (IP) receptor and constitutes the third major therapeutic pathway in PAH management [42]. Available formulations include oral agents (such as selexipag), inhaled preparations, subcutaneous infusions, and intravenous infusions, enabling stepwise therapeutic escalation based on disease severity and patient tolerability (Figure 2).

The GRIPHON trial was the first large-scale study to demonstrate that selexipag, an oral selective IP receptor agonist, reduced the risk of clinical deterioration or death by 40% (HR 0.60, *p* < 0.001), thereby contributing to improved long-term clinical outcomes [55].

The prevalence of ILD is high among patients with CTDs—approximately 30–60% in systemic sclerosis [56,57] and 30–78% in MCTD [58,59]. In pulmonary hypertension associated with ILD (group III), vasodilator therapy can exacerbate ventilation–perfusion (V/Q) mismatch [60,61]. Consequently, management has traditionally prioritized treatment of the underlying parenchymal lung disease. However, the 2021 INCREASE trial demonstrated that inhaled treprostinil significantly improved exercise capacity and reduced clinical worsening in ILD-associated PH [62] and provided therapeutic benefit without worsening V/Q mismatch. Notably, approximately 22% of enrolled patients had CTD-associated ILD, and the observed efficacy in this subgroup has important clinical implications. Similar benefits have been reported in Japan [63], positioning inhaled treprostinil as a promising and safe vasodilatory option for patients with CTD complicated by ILD.

Prostacyclin analogues and IP receptor agonists are associated with adverse effects such as headache, flushing, jaw pain, diarrhea, and hypotension. Parenteral formulations may additionally increase the risk of catheter-related infections and infusion-site complications.

### 4.2. Activin Signal Inhibitor (Sotatercept)

**Figure 3:**
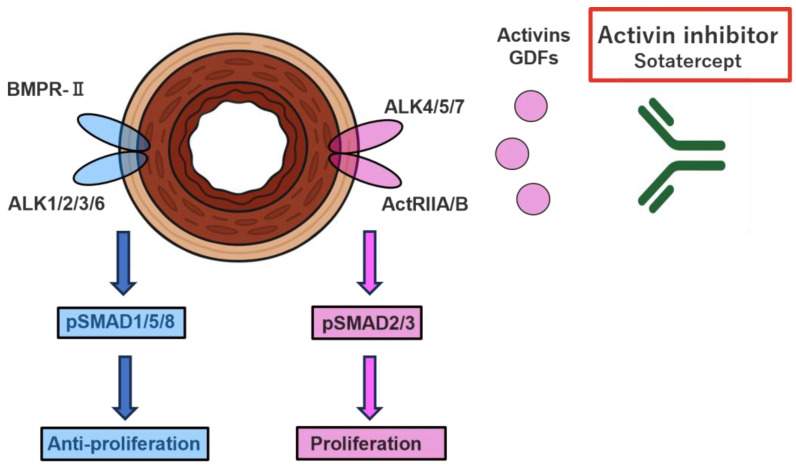
Mechanisms of action of sotatercept. Signaling abnormalities within the transforming growth factor-β (TGF-β) superfamily contribute to vascular remodeling in pulmonary arterial hypertension (PAH) by promoting smooth muscle cell proliferation. Activin and growth differentiation factor (GDF) ligands activate phospho-SMAD (pSMAD) 2/3 signaling through activin type II receptor (ActRIIA/B), whereas bone morphogenetic protein (BMP) ligands activate the pSMAD1/5/8 pathway via bone morphogenetic protein receptor type 2 (BMPR2). However, in PAH, activin signaling predominates. Sotatercept (activin receptor type IIA–Fc fusion protein, ActRIIA-Fc) selectively sequesters activin family ligands, thereby shifting the signaling balance toward antifibrotic activity and promoting reverse vascular remodeling.

Imbalances within the transforming growth factor (TGF)-β superfamily pathways have been implicated in the pathophysiology of PAH. The TGF-β family includes several ligands, such as TGF-β, activins, growth differentiation factors (GDFs), and bone morphogenetic proteins (BMPs). Activins, GDFs, and other TGF-β ligands bind to the human activin receptor type II (ActRIIA/B), thereby activating SMAD2/3 signaling and promoting downstream proliferative responses (Figure 3). In contrast, BMPs activate SMAD1/5/8 through the BMP receptor type II (BMPR2), exerting antiproliferative effects. In PAH, activin/SMAD2/3 signaling is upregulated relative to BMP/SMAD1/5/8 signaling, and mutations in BMPR2 have been implicated in disease susceptibility.

Sotatercept is a homodimeric recombinant fusion protein that functions as an inhibitor of activin signaling. Its structure comprises a modified extracellular domain of human activin receptor type IIa fused to a human IgG1 Fc domain (ActRIIA-Fc). By sequestering activins and other ligands of the TGF-β superfamily, sotatercept restores the balance toward the antiproliferative BMPR2/SMAD1/5/8 pathway [64]. Unlike conventional vasodilators, sotatercept is positioned as an anti-remodeling agent that directly targets vascular fibrosis.

In the 2023 STELLAR trial, sotatercept was administered in addition to background therapy in patients with PAH and produced simultaneous improvements across multiple endpoints, including 6 MWD, pulmonary vascular resistance, and NT-proBNP [65]. Furthermore, the 2025 ZENITH trial demonstrated that sotatercept reduced the risk of death, lung transplantation, and hospitalization in high-risk patients [66].

Anti-drug antibodies (ADAs) were detected in a proportion of treated patients; among 162 evaluable participants, 42 (25.9%) were ADA-positive through week 24, of whom 11 (6.8%) were also positive for neutralizing antibodies. To date, the presence of ADAs has not been associated with clinically meaningful effects on pharmacokinetics, efficacy, or safety [67]. However, given the relatively limited duration of follow-up, the long-term clinical significance of ADA development remains uncertain and warrants further investigation. Moreover, in light of the limited availability of CTD-specific data, the precise role of sotatercept in CTD-PAH—particularly across inflammation-dominant and fibrosis-dominant phenotypes—remains to be fully elucidated.

### 4.3. Immunosuppressive Agents

#### 4.3.1. Glucocorticoids (GC)

The therapeutic response to glucocorticoids (GCs) in CTD-PAH varies according to the underlying disease pathology. A French retrospective study of patients with MCTD demonstrated that approximately half of the cases could be adequately controlled with early initiation of GC-based immunosuppressive therapy [68]. Furthermore, in SLE-PAH and MCTD-PAH, the combination of high-dose GCs with additional immunosuppressive agents has been reported to achieve rapid clinical stabilization [69]. In contrast, the response to GCs in SSc-PAH is limited, likely due to the predominance of fibrotic and vaso-occlusive vascular lesions in systemic sclerosis [69].

Several Japanese cohort studies found that, among patients with PAH associated with SLE, MCTD, or Sjögren disease, combination therapy consisting of GCs (prednisolone 0.5 to 1 mg/kg/day) and immunosuppressive agents such as cyclophosphamide resulted in hemodynamic improvement and enhanced long-term prognosis [70,71]. The therapeutic effect was particularly notable in cases in which CTD and PAH were diagnosed simultaneously [70,71].

Overall, PAH associated with SLE and MCTD is strongly immune-mediated, and early GC-based immunosuppressive therapy can be effective. In contrast, SSc-PAH, characterized predominantly by progressive fibrosis, exhibits poor responsiveness to GC therapy.

#### 4.3.2. Cyclophosphamide

Cyclophosphamide (CY), an alkylating agent, exerts antitumor and immunosuppressive effects by inducing DNA crosslinking, thereby inhibiting cell division and promoting apoptosis [72]. In the context of CTD-PAH, CY has been shown to be particularly effective in inflammation-driven disease processes such as those seen in SLE- and MCTD-associated PAH, and is regarded as a valuable glucocorticoid-sparing agent [70,71]. In contrast, although CY has demonstrated therapeutic benefit for interstitial lung disease in systemic sclerosis [73], its efficacy for SSc-PAH remains limited.

CY is also associated with characteristic adverse effects, including carcinogenic risk such as bladder cancer, hematologic toxicity, and reduced fertility, which are not infrequently encountered in clinical practice. For younger patients with SLE or MCTD, in particular, the need for alternative therapeutic agents is increasingly recognized.

#### 4.3.3. B Cell Depletion Therapy (Rituximab)

Rituximab (RTX), an anti-CD20 monoclonal antibody, binds specifically to the CD20 antigen expressed on the surface of B cells and induces B-cell depletion through antibody-dependent cellular cytotoxicity (ADCC), complement-dependent cytotoxicity (CDC), and apoptosis [74].

The efficacy of rituximab in improving skin fibrosis and pulmonary function in systemic sclerosis has been demonstrated [75]. In SSc-PAH, abnormalities in autoantibody production and B-cell maturation—such as reduced usage of the IGHV2-5 gene segment—have been implicated in the development of vascular pathology. Notably, these molecular abnormalities have been reported to be partially normalized following B-cell depletion with rituximab [76].

A multicenter, double-blind, randomized controlled trial conducted in the United States in 2021 demonstrated encouraging findings for SSc-PAH. Although the primary endpoint was not met, secondary analyses revealed a significant improvement in 6 MWD through 48 weeks in the rituximab group compared with placebo [77]. Further investigation in larger, well-powered clinical trials is warranted.

#### 4.3.4. IL-6 Inhibitors (Tocilizumab, Satralizumab)

Interleukin-6 (IL-6) plays a pivotal role not only in immune cell activation and inflammatory signaling but also in promoting the proliferation and remodeling of pulmonary vascular smooth muscle cells and endothelial cells, as well as enhancing anti-apoptotic mechanisms. Elevated serum IL-6 levels are independently associated with reduced pulmonary function, progression of right heart failure, and increased mortality risk, functioning as a prognostic biomarker rather than a simple indicator of inflammation [78].

Although several case reports and small series have suggested the potential efficacy of tocilizumab, an IL-6 receptor–blocking antibody, in CTD-PAH [79], large-scale clinical evidence remains limited. Ongoing prospective trials, such as the SATISFY-JP study, are currently evaluating its therapeutic effectiveness [80]. SATISFY-JP is an investigator-initiated clinical trial using satralizumab, another IL-6 receptor–targeting antibody, designed to prospectively assess its efficacy—particularly in patients with an immune-responsive phenotype characterized by elevated IL-6 levels, who are considered the most suitable target population for this therapeutic approach.

#### 4.3.5. Mycophenolate Mofetil

Mycophenolate mofetil (MMF) inhibits inosine monophosphate dehydrogenase (IMPDH), thereby suppressing lymphocyte proliferation and inducing apoptosis, which leads to depletion of CD4^+^ and CD8^+^ T cells as well as B cells. This immunomodulatory effect is accompanied by an increase in regulatory T cells (Tregs), resulting in a more suppressive immune milieu [81].

**Figure 4:**
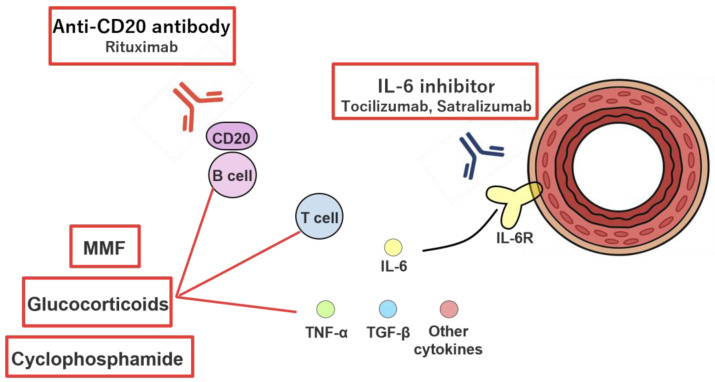
Summary of the mechanisms of action of various immunosuppressive therapies. In connective tissue disease-associated pulmonary arterial hypertension (CTD-PAH), activation of B lymphocytes and T lymphocytes initiates acute inflammation and subsequently promotes vascular smooth muscle cell proliferation through the production of autoantibodies and pro-inflammatory cytokines, including tumor necrosis factor-α (TNF-α) and transforming growth factor-β (TGF-β). Interleukin-6 inhibitors exert anti-inflammatory effects through selective blockade of the inflammatory cytokine interleukin-6, thereby suppressing the proliferation of vascular smooth muscle cells and endothelial cells and inducing apoptosis. Rituximab partially normalizes dysregulated immunologic pathways by selectively depleting CD20-positive B lymphocytes. Glucocorticoids, cyclophosphamide, and mycophenolate mofetil (MMF) suppress the proliferation of T lymphocytes and B lymphocytes and consequently attenuate excessive cytokine activity. MMF may also be associated with an increase in regulatory T lymphocytes, contributing to additional immunomodulatory effects.

In SSc, the Scleroderma Lung Study demonstrated that MMF has efficacy comparable to CY in treating skin fibrosis and ILD [73]. However, evidence regarding its effects on PAH remains limited. A recent single-center retrospective study reported that early initiation of MMF significantly reduced the risk of developing SSc-PAH (odds ratio 0.12, *p* = 0.048) [82], suggesting its potential role in preventing progression from inflammatory endothelial injury to fibrotic vascular remodeling. Figure 4 summarizes the currently postulated mechanisms of action of various immunosuppressive therapies for PAH.

Immunosuppressive therapies carry agent-specific risks, including infection, cytopenia, intestinal perforation, and other organ damage, necessitating careful patient selection and close monitoring.

### 4.4. Treatment Algorithm in CTD-PAH

Treatment strategies for CTD-PAH should follow a structured, risk-based, and phenotype-informed algorithm, as proposed in recent international guidelines [2]. After confirmation of PAH by right heart catheterization, the presence or absence of cardiopulmonary comorbidities—particularly ILD or left heart involvement—should be carefully assessed, as this distinction critically influences initial therapeutic choices.

In patients with CTD-PAH and significant cardiopulmonary comorbidities, current recommendations support initiating treatment with oral monotherapy using either a PDE5i or an ERA, regardless of baseline risk category. In contrast, in patients without cardiopulmonary comorbidities, treatment should be stratified according to baseline risk assessment. Patients classified as high risk should receive upfront triple combination therapy, including an ERA, a PDE5i, and a prostacyclin analogue. Patients at low or intermediate risk are recommended to start with initial dual oral combination therapy using an ERA and a PDE5i. Subsequent treatment escalation should be guided by regular follow-up risk reassessment, with the goal of achieving and maintaining a low-risk profile. In patients who fail to reach treatment goals, sequential intensification with additional prostacyclin pathway agents or emerging anti-remodeling therapies should be considered.

Importantly, immunosuppressive therapy represents an additional disease-specific component of the treatment hierarchy in selected CTD-PAH phenotypes. Inflammation-dominant forms, particularly SLE–PAH and MCTD–PAH, may benefit from immunosuppressive regimens, most commonly combining GCs with cyclophosphamide, in conjunction with PAH-targeted vasodilator therapy. In contrast, immunosuppressive strategies appear to have limited efficacy in fibrosis-dominant phenotypes such as SSc–PAH, where vasodilatory and anti-remodeling approaches remain the cornerstone of therapy.

## 5. Potential Biomarker for CTD-PAH

CTD-PAH encompasses a wide spectrum of underlying pathophysiological mechanisms and frequently presents with overlapping phenotypes, including concomitant left heart disease or ILD. Accordingly, there is a substantial unmet need for biomarkers that can facilitate early detection, refine risk stratification, and help distinguish immune-driven from fibrosis-dominant disease phenotypes. Such biomarkers may also serve as minimally invasive tools for monitoring disease activity, assessing treatment response, and guiding personalized therapeutic strategies in CTD-PAH.

However, the current evidence base for biomarkers in CTD-PAH remains limited and heterogeneous. Therefore, the following categorization is based on the primary potential clinical utility of each biomarker rather than definitive or exclusive roles. Importantly, several candidates may span multiple categories, reflecting both the biological complexity of CTD-PAH and the exploratory nature of biomarker research in this field.

Established biomarkers used in clinical practice include circulating cytokines such as interleukin-6 (IL-6), disease-specific autoantibodies—including anticentromere and anti–topoisomerase I antibodies—and cardiac biomarkers such as BNP and NT-proBNP [42]. In addition, several novel biomarker candidates with potential diagnostic, prognostic, or therapeutic relevance have been reported in recent years.

### 5.1. Biomarkers for Early Screening/Risk Stratification

Biomarkers that correlate with disease presence, severity, or hemodynamic burden may aid in early detection and risk assessment of CTD-PAH. Emerging evidence suggests that enhanced Th17 cell activity contributes to pulmonary vascular dysfunction and the development of PAH [83]. In SSc, IL-17A–positive patients exhibit elevated levels of inflammatory cytokines—including IL-1β, IL-6, and IL-22—together with reduced pulmonary function and a higher prevalence of PAH [84]. Furthermore, plasma proteomic analyses in high-risk PAH populations have demonstrated activation of the IL-17A–driven NF-κB pathway and the IL-6–driven JAK/STAT pathway, suggesting that simultaneous targeting of the IL-17A/IL-6 signaling axis may represent a promising future therapeutic approach [85]. These findings suggest that immune-related biomarkers may have utility in identifying patients at increased risk for CTD-PAH, although their role in formal screening strategies requires further validation.

### 5.2. Biomarkers Reflecting Immune Activity and Treatment Responsiveness: CTRP7

CTRP7 (C1q/TNF-related protein 7) has recently been proposed as a biomarker with potential relevance to treatment response. CTRP7 expression is markedly upregulated in pulmonary arterial smooth muscle cells from patients with PAH and in experimental animal models, and circulating plasma levels are elevated compared with healthy controls [86]. Mechanistically, IL-6 promotes CTRP7 transcription, which in turn enhances the internalization and degradation of the prostacyclin receptor (PTGIR) via Rab5a, leading to reduced responsiveness to selexipag [86]. These findings suggest that CTRP7 may serve as a biomarker predictive of prostacyclin pathway efficacy. Although CTRP7 has previously been linked to atherosclerosis and insulin resistance [87,88], its clinical relevance in CTD-PAH has not yet been explored, underscoring the need for disease-specific investigation.

### 5.3. Biomarkers Indicative of Fibrotic and Vascular Remodeling: HIF-1α and VEGF

Hypoxia-inducible factor-1α (HIF-1α) is a transcription factor activated under hypoxic conditions that induces the expression of vascular endothelial growth factor (VEGF) and promotes angiogenesis. Serum concentrations of HIF-1α and VEGF are significantly elevated in patients with CTD-PAH compared with patients with CTD alone and healthy controls, and these levels correlate with pulmonary arterial pressure, right ventricular workload, and 6 MWD [89]. These associations suggest that HIF-1α and VEGF may reflect the extent of vascular remodeling and hypoxia-related disease burden. As such, they may function as surrogate markers of fibrotic or remodeling-dominant pathology, although their specificity for CTD-PAH remains to be established.

### 5.4. Biomarkers of Fibrotic Burden: Human Epididymis Protein 4

Human epididymis protein 4 (HE4) is a glycoprotein initially identified as a biomarker of fibrosis in gynecologic malignancies, particularly ovarian cancer [90], and has more recently attracted attention in non-neoplastic diseases. In IgG4-related disease, HE4 has been reported to independently predict organ fibrosis, visceral involvement, and poor prognosis [91]. In addition, in idiopathic PAH, elevated HE4 levels have been associated with disease severity and progressive cardiac functional decline [92]. Although HE4 has not yet been evaluated in CTD-PAH, these findings raise the possibility that it may serve as a marker of fibrotic burden and irreversible vascular remodeling in this disease context.

## 6. Animal Models for CTD-PAH

There is a critical need to develop robust animal models to advance therapeutic research and elucidate the underlying pathophysiology of CTD-PAH. The disease arises from a complex interplay of immune dysregulation, vascular remodeling, and fibrosis—processes that cannot be fully recapitulated through in vitro systems or human observational studies alone. Moreover, the heterogeneity of CTD-PAH, including the coexistence of inflammatory and fibrotic phenotypes and overlap with Group II and Group III mechanisms, underscores the necessity of experimental models that can dissect disease-specific pathways. Animal models are also indispensable for validating emerging biomarkers and for preclinical evaluation of novel therapies such as immune-targeted agents and anti-remodeling drugs. To date, several mouse models that recapitulate key features of CTD-PAH have been reported.

### 6.1. TNF-Transgenic Mouse Model

TNF-transgenic (TNF-Tg) mice are a transgenic model in which the human TNF-α gene is overexpressed. Notably, female mice develop pulmonary vascular remodeling characterized by collagen deposition, endothelial injury, and smooth muscle cell proliferation within the pulmonary vasculature, leading to elevated right ventricular pressures and right ventricular hypertrophy [93]. Furthermore, inhibition of TNF-α has been shown to ameliorate vascular lesions in this model [93], indicating that the pathological changes remain at least partially reversible during the inflammatory stage.

Recent work has further clarified the mechanisms by which TNF overexpression drives pulmonary vascular disease in this model [94]. Single-cell RNA sequencing revealed a marked loss of microvascular endothelial subsets, expansion of proliferating endothelial and smooth muscle cells, and near-complete depletion of pericytes, reproducing key structural abnormalities observed in PAH. The study also demonstrated a shift in fibroblast phenotype toward matrix-producing populations, accompanied by excessive basement membrane protein deposition. Importantly, TNF signaling was shown to induce profound dysregulation of the BMP pathway, characterized by loss of BMPR2 expression and aberrant BMP2-driven signaling, leading to SMAD2/3 activation, endothelial injury, and smooth muscle cell proliferation. These findings highlight that TNF-Tg mice recapitulate not only the inflammatory milieu but also the aberrant BMP signaling network fundamental to PAH, underscoring the value of this model for mechanistic studies and for evaluating therapies targeting TNF–NF-κB or BMP pathway restoration [94].

### 6.2. Pristane/Hypoxia (PriHx) Model

The pristane/hypoxia (PriHx) model is a composite disease model that integrates SLE-like autoimmune abnormalities induced by pristane administration with hypoxia-driven vascular injury and proliferative stimulation [95]. In this model, immune cell infiltration, autoantibody production, vascular remodeling, fibrosis, and right ventricular hypertrophy occur concurrently, thereby recapitulating the continuum from immune dysregulation to fibrotic pathology—a progression that has been difficult to reproduce using single-factor models.

### 6.3. MRL/lpr Mouse Model

MRL/lpr mice represent a model of severe SLE-like autoimmunity in which autoreactive lymphocytes persist and accumulate due to Fas gene mutation [96]. In this model, not only pulmonary vascular abnormalities but also pronounced right ventricular pathology—including chamber enlargement, fibrosis, and functional impairment—are observed. This model is therefore unique in its ability to recapitulate the pathological progression from pulmonary vascular injury to right-sided heart failure.

### 6.4. Fra-2-Transgenic Mouse and PSGL-1-Deficient Mouse Model

Other models have also been developed to elucidate various aspects of CTD-PAH. These include Fra-2 transgenic mice, which display SSc–like pathology [16], and *p*-selectin glycoprotein ligand 1 (PSGL-1)–deficient mice, which spontaneously develop SSc-like autoimmune abnormalities and PAH [97]. Collectively, these models capture distinct and complementary features of CTD-PAH and serve as valuable research tools for advancing mechanistic understanding and therapeutic development.

Importantly, these emerging biomarkers and experimental models should not be viewed solely as mechanistic tools but as potential bridges between molecular pathology and clinical phenotypes. By reflecting immune activation, vascular remodeling, or fibrotic burden, such markers and animal models may ultimately support phenotype-guided therapy selection and treatment monitoring in CTD-PAH.

## 7. Unmet Needs in the Treatment of CTD-PAH

CTD-PAH requires diagnostic and therapeutic strategies tailored to its diverse underlying diseases and complex pathophysiology. Although advances in vasodilator therapy, immune-targeted treatments, and anti-remodeling agents have been made, substantial gaps in evidence and clinical practice remain. These unmet needs delay early diagnosis, limit individualized therapy, and contribute to poor long-term outcomes. Importantly, deeper elucidation of the disease’s underlying pathobiology is essential for addressing these challenges and for enabling more precise classification and targeted treatment development. Clarifying these issues is critical for guiding future research priorities and improving patient care. The principal issues are outlined below.

### 7.1. Lack of Disease-Specific Treatment Strategies

CTD-PAH encompasses a heterogeneous group of underlying conditions, including SSc, SLE, MCTD, and others, each of which is characterized by distinct pathophysiological mechanisms. Despite these differences, disease-specific therapeutic approaches remain limited, and current management relies largely on vasodilator therapies analogous to those used for idiopathic PAH. In particular, patients with SSc-PAH often present at a stage when irreversible fibrosis has already developed, and immunosuppressive therapy is generally ineffective, resulting in missed opportunities for early intervention. This delay in treatment initiation is considered a major contributor to the poor prognosis associated with SSc-PAH.

### 7.2. Lack of Adequate Biomarkers for Precision Medicine

Indicators for assessing disease activity and therapeutic response in CTD-PAH remain markedly insufficient. Recently identified biomarker candidates—such as CTRP7 and HE4—have garnered attention as potential indicators of prostacyclin receptor sensitivity and fibrotic burden; however, their clinical applicability in CTD-PAH has not yet been established. Rigorous validation, assay standardization, and integration into longitudinal clinical cohorts will be essential to determine their diagnostic and prognostic utility. Furthermore, existing biomarkers such as IL-6, BNP, and NT-proBNP lack disease specificity and cannot reliably distinguish pre-capillary PAH from PH due to left heart disease or ILD in patients with CTD. The absence of robust, disease-relevant biomarkers not only hampers early detection but also limits the development of phenotype-guided and mechanism-targeted therapeutic strategies—an essential step toward true precision medicine in CTD-PAH.

### 7.3. Lack of Evidence for Therapeutic Agents in CTD-PAH

Although emerging therapies—including rituximab, IL-6 receptor inhibitors, and the activin pathway inhibitor sotatercept—have demonstrated encouraging clinical signals, large-scale randomized controlled trials specifically targeting CTD-PAH have not yet been conducted. Consequently, the evidence base remains insufficient to determine optimal timing, patient selection, or combination strategies for these agents. Notably, no definitive treatment algorithms exist for CTD-PAH, and clinical decision-making continues to rely largely on empirical experience rather than evidence-based guidelines. The heterogeneity of CTD-PAH—ranging from inflammation-dominant to fibrosis-dominant disease—further complicates therapeutic evaluation, as treatment response differs substantially across phenotypes. Without disease-specific clinical trials, it remains challenging to establish precision therapies that address the unique immunologic, vascular, and fibrotic mechanisms present in each CTD subtype.

## 8. Conclusions

CTD-PAH is a severe and heterogeneous form of pulmonary hypertension that arises in the context of systemic autoimmune disease and requires disease-tailored diagnostic and therapeutic approaches. This review has summarized the current epidemiology, pathophysiology, available and emerging therapies, biomarker development, and relevant animal models of CTD-PAH.

Clinical characteristics, treatment responsiveness, and prognosis vary substantially across CTD subtypes, with SSc-PAH consistently demonstrating the poorest outcomes. Although vasodilator therapies targeting the endothelin, nitric oxide, and prostacyclin pathways remain the cornerstone of treatment, accumulating evidence suggests that emerging agents—such as the activin signal inhibitor sotatercept, B-cell–targeted therapy with rituximab, and IL-6 pathway inhibitors—may provide additional benefit in selected disease phenotypes. In parallel, advances in biomarker research and experimental animal models are improving early detection, risk stratification, and mechanistic understanding of immune-mediated and fibrotic vascular remodeling.

Looking ahead, future research should focus on the development of personalized treatment strategies based on integrated molecular, immunologic, and phenotypic profiling. Combination approaches that simultaneously target immune dysregulation and fibrotic vascular remodeling may be particularly relevant for addressing the heterogeneous disease spectrum of CTD-PAH. In addition, the management of CTD-associated ILD–PH overlap syndromes remains a major clinical challenge and warrants dedicated prospective clinical trials. Ultimately, the integration of validated biomarkers, advanced imaging modalities, and mechanistic insights derived from animal models will be essential for advancing precision medicine and improving long-term outcomes in patients with CTD-PAH.

## Data Availability

No new data were created or analyzed in this study.
